# Neuropsychiatric Symptoms in Huntington’s Disease: A Case Report on Manic and Psychotic Features Huntington’s disease (HD)

**DOI:** 10.1192/j.eurpsy.2025.534

**Published:** 2025-08-26

**Authors:** S. Buyo Lagares, A. Picallo Vieito, M. Grueiro Cao, M. Pardal Iglesias, A. Lagoa Pena, C. Ramil López, C. Ovies Fernández, A. Parada Barcia, P. Piñeiro Magro, L. González Pereira, J. Ricoy Chaín, R. Prieto Pérez

**Affiliations:** 1 Hospital A Coruña, A Coruña, Spain

## Abstract

**Introduction:**

Huntington’s disease (HD) is a hereditary neurodegenerative disorder marked by progressive declines in motor, cognitive, and psychiatric functions. This case report presents a 45-year-old female patient with HD who displayed significant manic symptoms, which later evolved into acute psychosis. Notably, her neuropsychiatric symptoms emerged months before motor deficits. This case aims to raise clinician awareness of the interplay between neuropsychiatric symptoms and HD.

**Objectives:**

Analyze early neuropsychiatric manifestations, particularly manic and psychotic symptoms; highlight the importance of recognizing these symptoms before the onset of motor dysfunction; and explore the neurobiological mechanisms underlying, including neurotransmitter dysregulation and structural brain changes.

**Methods:**

A comprehensive clinical evaluation was conducted for the patient. Her psychiatric history was assessed using standardized tools, including the Young Mania Rating Scale (YMRS) and the Positive and Negative Syndrome Scale (PANSS).

Neuroimaging, including computed tomography (CT), assessed structural brain changes in regions related to mood regulation and psychosis, such as the striatum and prefrontal cortex. A literature review correlated these findings with existing research on neurobiological mechanisms in HD, focusing on neurotransmitter systems and brain morphology.

**Results:**

Initially, the patient exhibited manic symptoms such as elevated mood and irritability, with moderate severity noted on the YMRS. Within a month, her condition escalated to acute psychosis, featuring auditory hallucinations and paranoid delusions, as reflected by moderate PANSS scores. Neuroimaging revealed structural changes consistent with HD, including striatal atrophy and prefrontal cortex alterations. These findings supported the hypothesis of neurotransmitter dysregulation, particularly involving dopamine and serotonin.

The management plan included mood stabilizers and antipsychotics, such as valproic acid and risperidone, along with temporary benzodiazepines to manage agitation. This approach led to a significant reduction in both manic and psychotic symptoms, improving the patient’s overall quality of life through integrated psychiatric care.

**Conclusions:**

This case underscores the importance of recognizing early neuropsychiatric symptoms, particularly manic and psychotic features, in HD patients. The emergence of these symptoms prior to motor dysfunction calls for heightened clinician awareness, as early identification can facilitate timely interventions and enhance patient outcomes. The observed structural brain changes and neurotransmitter dysregulation suggest underlying neurobiological mechanisms, warranting further research in the broader HD population. A multidisciplinary approach is essential for effectively managing the interplay of neuropsychiatric symptoms.

**Disclosure of Interest:**

None Declared

